# The Longitudinal Associations of Self-Perceptions of Aging and the Age Composition of Social Networks

**DOI:** 10.1093/geroni/igaa057.2109

**Published:** 2020-12-16

**Authors:** Ella Cohn-Schwartz, Markus Schafer, Liat Ayalon

**Affiliations:** 1 Ben Gurion University, Haifa, Israel; 2 University of Toronto, Toronto, Ontario, Canada; 3 Bar-Ilan University, Ramay Gan, HaMerkaz, Israel

## Abstract

Relying on the age segregation theory (limited contact between the generations), this study examined the temporal associations between the age composition of one’s social ties and one’s self-perceptions of aging (SPA). Data came from the 2014 and 2017 waves of the German Ageing Survey (DEAS). Age composition of the network was assessed as the number of kin and non-kin in the social network who are either five years older or five years younger than the respondent. A latent change score model assessed the bidirectional associations. Adults who had younger social network members, both kin and non-kin, had better SPA three years later. A positive SPA at baseline also predicted a higher number of younger non-kin relationships over time. These results stress the role of SPA in adults’ social network as well as the role of the age of social network members in shaping adults’ SPA.

